# Impact of Reducing Agents on Protein Synthesis in a Reconstituted Cell-Free Protein Synthesis System

**DOI:** 10.1021/acssynbio.6c00011

**Published:** 2026-02-17

**Authors:** Tomoe Fuse-Murakami, Shohei Terazawa, Riddhi Gondhalekar, Shohei Ito, Seiichi Miyawaki, Yusuke Mizukami, Willian P. Salgado, Zening Yang, Kosuke Fujishima, Takashi Kanamori

**Affiliations:** † GeneFrontier Corporation, 273-1 Kashiwa, Kashiwa, Chiba 277-0005, Japan; ‡ School of Life Science and Technology, 13290 Institute of Science Tokyo, Tokyo 152-8550, Japan; # School of Science, Institute of Science Tokyo, Tokyo 152-8550, Japan; ∥ School of Materials and Chemical Technology, Institute of Science Tokyo, Tokyo 152-8550, Japan; ⊥ Earth-Life Science Institute (ELSI), Institute of Science Tokyo, Tokyo 152-8550, Japan

**Keywords:** cell-free protein synthesis, PURE system, reducing agent, chelator

## Abstract

Maintaining proper redox conditions is essential for protein stability and function. In cell-free protein synthesis, reducing agents, such as dithiothreitol and reduced glutathione, are commonly added to mimic the cytosolic environment and prevent unwanted oxidation. The PURE system, which is a fully reconstituted protein synthesis system, also contains reducing agents. Here, we systematically examined how reducing agents affect the protein synthesis in the PURE system. We found that the reducing activity of dithiothreitol decreased during prolonged reactions, leading to the formation of disulfide bonds in synthesized proteins. Dissolved oxygen and contaminating metal ions were identified as major factors causing this loss of activity. Based on these findings, we developed a method to maintain reducing conditions throughout the reaction, ensuring consistent protein quality. Our results provide new insights into redox regulation in cell-free systems and offer a practical strategy for the efficient synthesis of functional proteins, with potential applications in biotechnology and therapeutic protein production.

## Graphical abstract

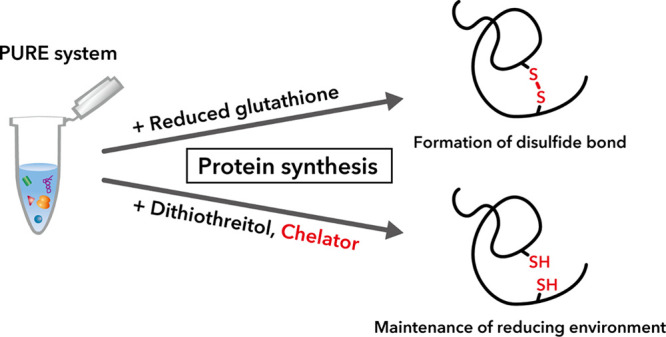

## Introduction

The structure and function of proteins are highly dependent on the surrounding redox environment. For example, the oxidation of cysteine residues leading to disulfide bond formation plays a critical role in protein stability and activity. In cells, the cytosol is maintained in a reducing state, whereas the endoplasmic reticulum (ER) in eukaryotes and the periplasm in prokaryotes are kept in an oxidizing state mainly through regulation of the glutathione concentration. Disruption of these redox environments is known to cause protein misfolding and functional impairment, which can severely affect cellular processes.^1–3^

Maintaining appropriate redox conditions is also important *in vitro*. For reactions involving cytosolic proteins, reducing agents such as dithiothreitol (DTT) and reduced glutathione (GSH) are commonly added to prevent oxidation. Cell-free protein synthesis (CFPS) systems enable protein production *in vitro* using cell extracts or reconstituted translation components.^4^ Since the translation reaction occurs in a cytosol-like environment, reducing agents are typically included in the reaction mixture.^4^ These systems can synthesize not only intracellular proteins but also extracellular proteins, such as antibodies. The PURE system, which consists solely of purified translation factors in *Escherichia coli*, lacks redox enzymes and thus allows precise control of the redox environment.^5^ Indeed, antibodies containing disulfide bonds can be synthesized with their activities in the PURE system simply by adding oxidized glutathione and appropriate molecular chaperones.^6,7^ Interestingly, the extent of disulfide bond formation in synthesized antibodies varies depending on the reducing agent used, even though translation itself is largely unaffected.^6^ This suggests that the activity and disulfide bond formation of the synthesized protein depend more on the presence of reducing agents than on the overall redox state. Moreover, DTT solutions are known to lose reducing activity over time during incubation.^8^ Since CFPS reactions typically run for extended periods, the decline in DTT activity during protein synthesis is a potential concern. The protein synthesis reaction using the current PURE system typically stops within approximately 6 h.^6,9^ However, in some cases, such as disulfide bond formation, a long incubation period after protein synthesis is required.^6^ Furthermore, continuous improvements of the PURE system have been reported,^10–12^ and further extension of reaction lifetimes is expected in the future.

In this study, we investigated the time-dependent decrease in the reducing activity of DTT during prolonged protein synthesis using the PURE system. We identified dissolved oxygen and contaminating metal ions as the primary factors responsible for this loss of activity. Based on these findings, we established a method using a chelator to maintain reducing conditions throughout the reaction. Our results provide new insights into redox regulation in cell-free systems and offer a practical strategy for the efficient synthesis of functional proteins.

## Results and Discussion

### Effect of Reducing Agents on Disulfide Bond Formation in the Synthesized Protein

First, we synthesized *E. coli* alkaline phosphatase (ALP), which contains two intramolecular disulfide bonds,^13^ using the PURE system in the presence of DTT or GSH to evaluate the effect of reducing agents on disulfide bond formation. When ALP was synthesized in the presence of GSH, the results under nonreducing conditions in Figure 1A,B indicate that ALP was predominantly synthesized in the oxidized state after 4 h. In contrast, using the reaction mixture containing DTT, the reduced form was detected up to 8 h, but the majority of the products was detected in the oxidized form at 24 h (Figure 1A–C). Since disulfide bonds are essential for ALP activity,^13^ we also measured the enzymatic activity using *p*-nitrophenol phosphate (PNPP). When synthesized in the presence of GSH, the activity was detected after 4 h. In contrast, in the reaction with DTT, activity was observed at 24 h (Figures 1D and S1). These results indicate that ALP synthesized in the PURE system is highly susceptible to oxidation, which cannot be suppressed by GSH. Moreover, DTT is also ineffective in maintaining a reduced environment during prolonged incubation.

**Figure 1:**
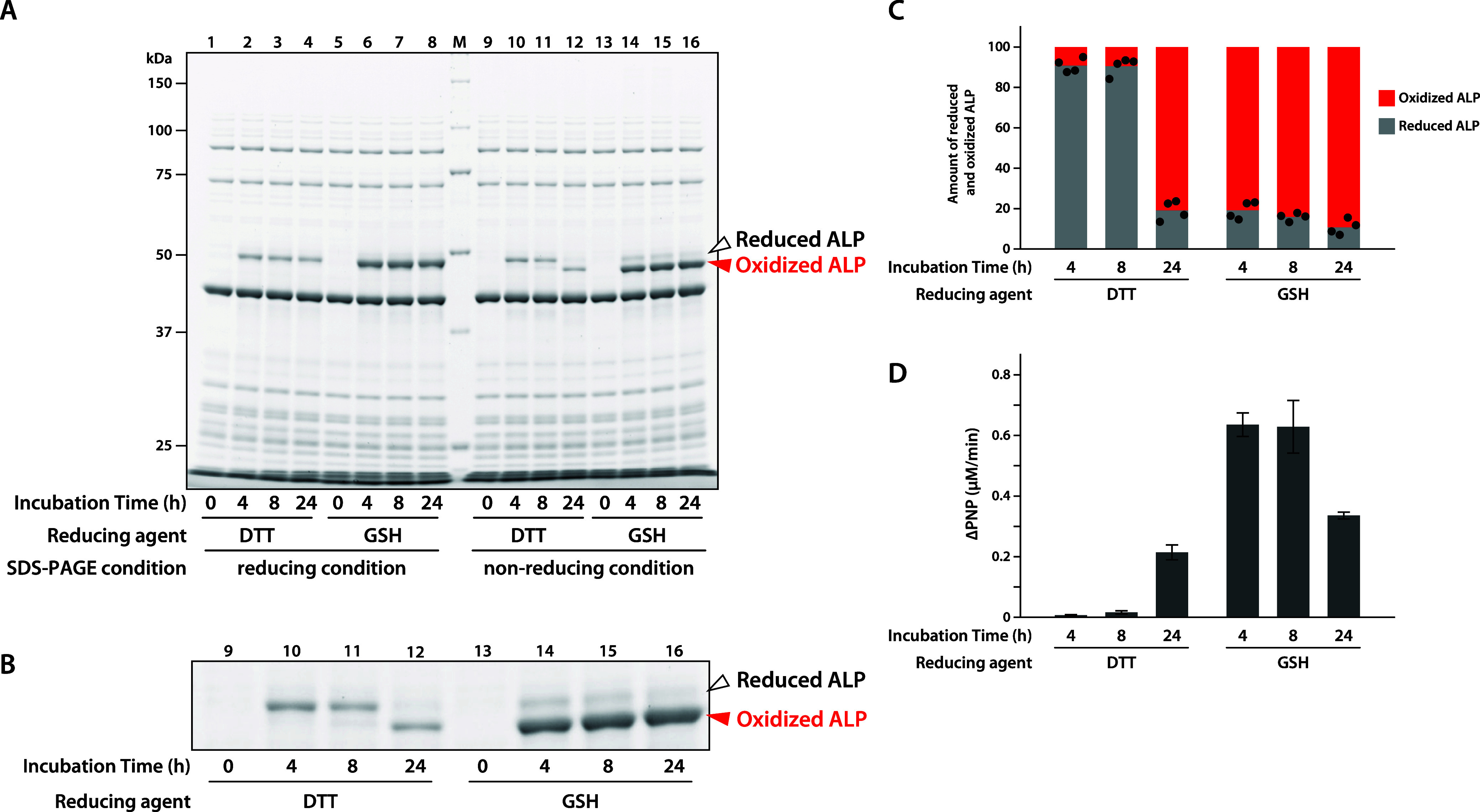
Synthesis of ALP in the presence of DTT or GSH. (A) *E. coli* ALP was synthesized using the PURE system in the presence of DTT or GSH at 37 °C for the indicated time. Reaction mixtures were subjected to SDS-PAGE under reducing or nonreducing conditions. Gels were stained with SYPRO Orange and visualized using the LuminoGraph imaging system. M: molecular weight marker. (B) Enlarged image of the region containing synthesized ALP under nonreducing conditions in (A). (C) Intensities of bands corresponding to reduced and oxidized forms of ALP under nonreducing SDS-PAGE conditions quantified using LuminoGraph. The ratio of reduced form (gray) to oxidized form (red) was calculated. Data represent averages from four independent experiments. (D) Enzyme activity of the synthesized ALP assessed using PNPP as a substrate. The amount of PNP produced by ALP-mediated dephosphorylation of PNPP was measured by the absorbance at 405 nm. The average increase in PNP per reaction mixture is shown. Data are presented as mean ± standard deviation from four independent experiments.

### Oxidation of DTT’s Sulfhydryl Groups Involves Contaminating Metal Ions and Dissolved Oxygen

ALP synthesized in the presence of DTT was found to be oxidized after 24 h of protein synthesis. We conducted a detailed investigation into the factors contributing to DTT instability under the reaction conditions. The reducing power of DTT was evaluated by quantifying the remaining sulfhydryl groups using 5,5′-dithiobis­(2-nitrobenzoic acid) (DTNB).^14^ First, DTT was incubated in the reaction mixture of the PURE system, excluding proteins and ribosomes. In this assay, the sulfhydryl groups of DTT were found to be completely depleted, suggesting that the depletion was due to components in the buffer (Figure 2A). Next, we performed a further investigation by incubating DTT separately with each component in the buffer. Because the sulfhydryl groups of DTT decreased even in the presence of HEPES–KOH alone (Figure 2A), we conducted this assay with or without HEPES–KOH to examine the influence of HEPES–KOH on each component. From these results, sulfhydryl groups of DTT were found to decrease even in the presence of potassium glutamate (KGlu) and magnesium acetate (Mg­(OAc)_2_) solution alone (Figure 2A). Oxidation of DTT by KGlu and Mg­(OAc)_2_ solution was consistently observed, even when reagents from different suppliers were used (Figure S2). This reaction proceeded linearly over time (Figure 2B), suggesting that DTT undergoes a chemical reaction with certain molecules in the buffer. In contrast, GSH retained its sulfhydryl groups, even when incubated in the reaction mixture containing buffer components (Figure S3).

**Figure 2:**
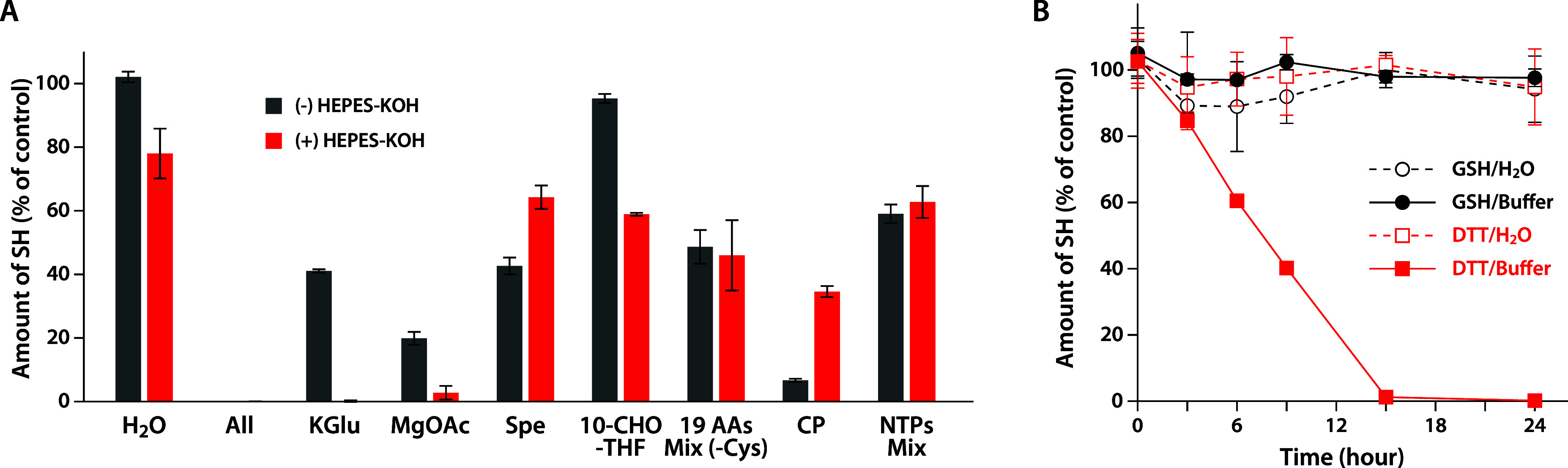
Oxidation of DTT. (A) DTT solution was incubated with each chemical component of the PURE system at 37 °C for 24 h either in the presence (red bars) or absence (black bars) of 10 mM HEPES–KOH (pH 7.6). After incubation, the remaining sulfhydryl groups were measured using DTNB, as described in Materials and Methods. The values are expressed as ratios relative to the initial amount of DTT without HEPES–KOH. Data represent mean ± standard deviation from three independent experiments. Spe, 10-CHO-THF, 19 AAs Mix (-Cys), CP, and NTPs Mix indicate spermidine, 10-formyltetrahydrofolate, 19 amino acid mixtures except cysteine, creatine phosphate, and ATP/GTP/CTP/UTP mixture, respectively. (B) Time course of oxidation of DTT and GSH. Samples were incubated at 37 °C for 0, 3, 6, 9, 15, and 24 h in either water or HKM buffer. DTT in water (red dotted line) and HKM buffer (red solid line) and GSH in water (black dotted line) and HKM buffer (black solid line) were analyzed. Sulfhydryl content was measured using DTNB, and values are shown as ratios relative to the initial amount in water. Data represent mean ± standard deviation from three independent experiments.

Because potassium and magnesium ion are indispensable components for protein synthesis using the PURE system,^5^ their exclusion is not feasible. Therefore, we explored strategies to preserve the reducing environment under these conditions. DTT is known to react with metal ions.^15^ Considering the possibility of trace metal ion contamination in Mg­(OAc)_2_ and KGlu, we conducted the same reaction in the presence of chelators (Figure 3A). When ethylenediaminetetraacetic acid (EDTA), a widely used chelator, was added, the oxidation of DTT’s sulfhydryl groups was suppressed, with approximately half of the sulfhydryl groups remaining. With diethylenetriaminepentaacetic acid (DTPA), a stronger chelator than EDTA, more than 80% of the SH groups were retained. Furthermore, upon addition of deferiprone (DFP), an iron-specific chelator, approximately half of the sulfhydryl groups of DTT were preserved, indicating that contaminating iron ions play a significant role in the oxidation of DTT. We further examined whether oxygen was involved in the oxidation of DTT. Under oxygen-depleted conditions, the remaining SH groups were higher than those under oxygen-rich conditions, even in the absence of DTPA (Figure 3B). These results indicate that the oxidation of DTT involves both contaminating metal ions and dissolved oxygen.

**Figure 3:**
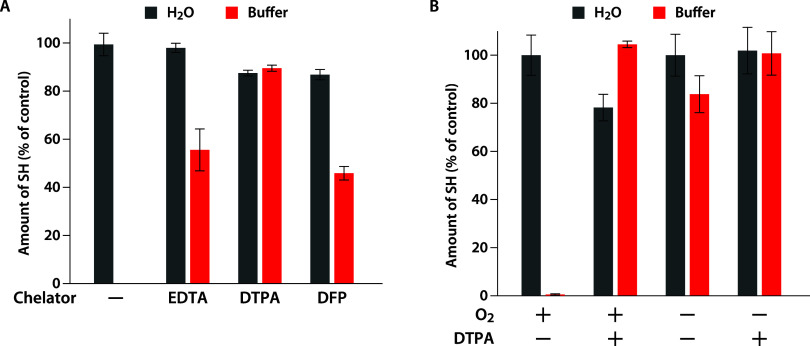
Suppression of DTT oxidation. (A) DTT was incubated at 37 °C for 24 h in either water (gray bars) or HKM buffer (red bars) in the presence of the indicated chelator. (B) DTT was incubated at 37 °C for 24 h either inside the glovebox (O_2_ −) or outside the glovebox (O_2_ +), with or without DTPA. After incubation, the remaining sulfhydryl groups were measured using DTNB. The results are expressed as ratios relative to the initial amount of DTT in water before incubation. Data represent mean ± standard deviation from three independent experiments.

### Protein Synthesis with Disulfide Bonds in the Presence of a Chelator

Complete removal of dissolved oxygen under standard laboratory conditions remains challenging. In contrast, the addition of a chelator is a simple and practical method. Common chelators such as EDTA and DTPA can also bind magnesium ions, which is essential for transcription and translation. To evaluate the impact of adding a chelator on protein synthesis, we synthesized *E. coli* dihydrofolate reductase (DHFR), which is commonly used as a model protein,^5^ in the presence of DTPA. The results showed that the addition of 2 mM DTPA did not significantly decrease the synthesis yield and the rate (Figure S4). When ALP was synthesized in the presence of DTT and DTPA, most of the synthesized product remained in its reduced form (Figure 4A). The synthesized ALP in the presence of DTT and DTPA showed no activity (Figures 4B and S5A). These findings suggest that the reaction mixture containing both DTT and DTPA effectively maintains a reducing environment without compromising the protein synthesis efficiency. The affinity of DTPA for magnesium is substantially lower than that for iron and copper. The stability constants (log *K*) of DTPA with magnesium, iron and copper are 9.3, 27.3, and 21.5, respectively.^16^ This large difference in stability constants indicates that at low chelator concentrations DTPA preferentially binds iron and copper rather than magnesium. Therefore, low concentrations of chelators exert minimal influence on the translation efficiency.

**Figure 4:**
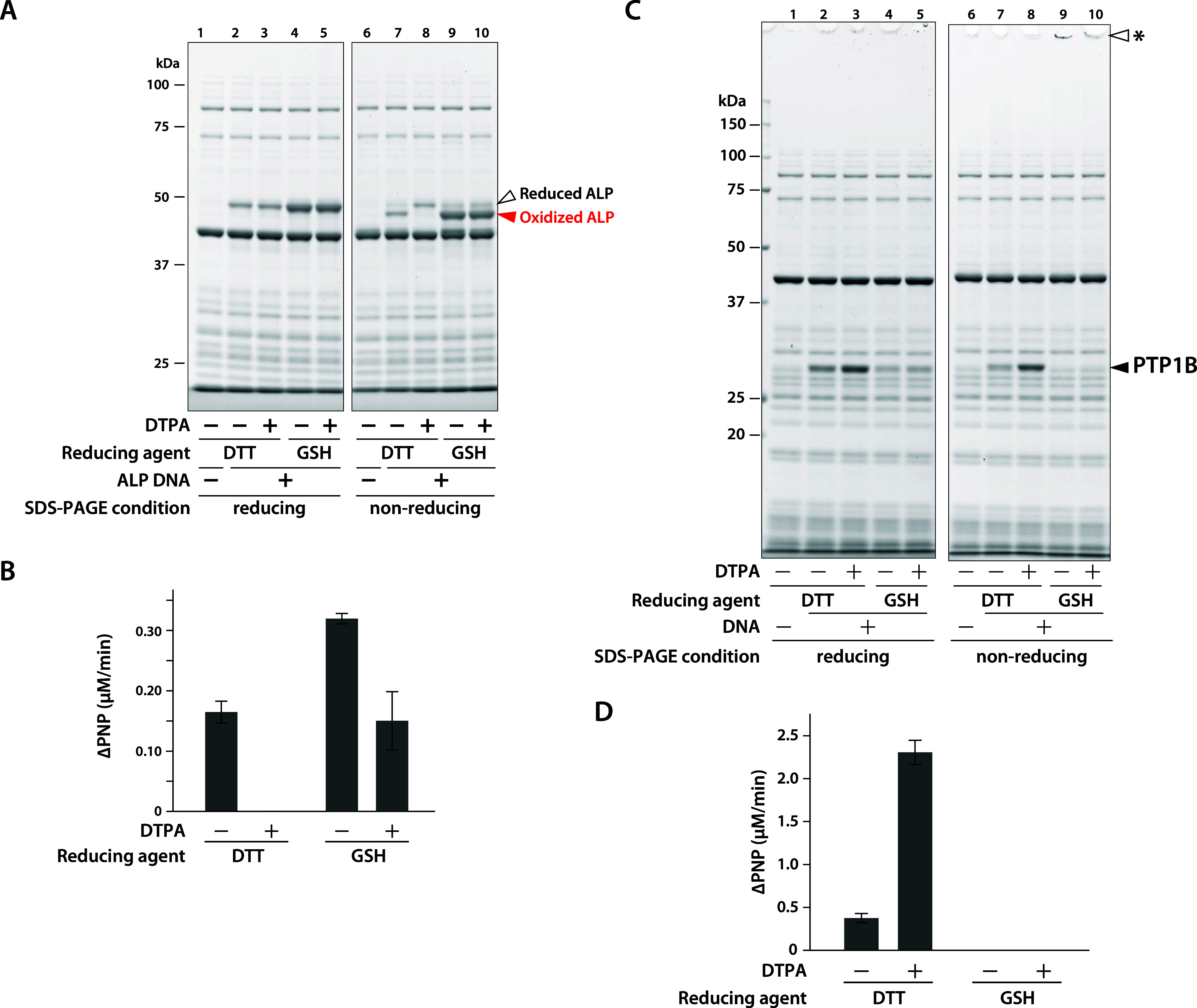
Protein synthesis in the presence of DTPA. (A) ALP or (B) PTP1B was synthesized using the PURE system at 37 °C for 24 h in the presence of either DTT or GSH, with or without DTPA. Reaction mixtures were analyzed by SDS-PAGE under reducing and nonreducing conditions. Gels were stained with SYPRO Orange and visualized using LuminoGraph. An asterisk in B indicates aggregated products. (C, D) Enzymatic activity of the synthesized (C) ALP or (D) PTP1B was measured by PNPP phosphatase assay. The amount of PNPP hydrolyzed per reaction mixture is shown. Data represent mean ± standard deviation from three independent experiments.

In contrast, when GSH was used, only the oxidized form of ALP was synthesized, regardless of the presence of DTPA. In contrast, the enzymatic activity of ALP decreased. Since zinc ions are essential for ALP activity,^17^ this decrease is likely due to chelation of zinc ions by DTPA rather than reduction of the disulfide bond. While the redox potential of DTT is −330 mV and that of GSH is −240 mV at pH 7.0, indicating that GSH is a weaker reducing agent, its reducing potential is still sufficient to suppress disulfide bond formation.^18–20^ However, GSH did not inhibit the disulfide bond formation of the synthesized proteins. Because glutathione can reversibly shift between reduced and oxidized states depending on the environmental conditions, it is possible that glutathione reached equilibrium under the conditions of this experiment and therefore neither inhibited the formation of disulfide bonds in the synthesized products nor reduced preformed disulfide bonds. This hypothesis requires further experimental validation.

### Reductive Synthesis of Functional Proteins with DTT and DTPA

Finally, we synthesized a protein whose activity depends on maintaining a reducing environment. Protein tyrosine phosphatase PTP1B requires the cysteine residue at its active site to remain in a reduced state.^21^ When PTP1B was synthesized in the presence of DTPA, enzymatic activity was detected with DTT, whereas no activity was observed with GSH (Figures 4D and S5B). In SDS-PAGE analysis under nonreducing conditions, no band corresponding to the expected molecular weight was detected with GSH; instead, a band was observed near the bottom of the well (Figure 4C). Furthermore, by centrifugation of the reaction mixture, most of the product was recovered in the supernatant when DTT was used, while no soluble product was recovered with GSH (Figure S6). Additionally, we tested tris­(2-carboxyethyl)­phosphine (TCEP) as an alternative to DTT. TCEP is a thiol-free reducing agent that has been used previously in the PURE system.^6^ When ALP was synthesized in the presence of TCEP, it was obtained predominantly in a reduced form, even in the absence of DTPA. In addition, the enzymatic activity of the synthesized PTP1B increased to a similar extent as observed with DTT (Figure S7). These results indicate that maintaining a reducing environment with DTT or TCEP and DTPA is crucial for synthesizing active PTP1B.

## Conclusion

When cell extracts are used, the redox state is influenced by endogenous redox enzymes, making it difficult to control solely with added reducing agents. In contrast, the PURE system, being a reconstituted system, lacks such enzymes and allows for precise control of the redox environment through the addition of reducing agents. Our findings demonstrate that the reaction conditions can be flexibly optimized not only for the synthesis of proteins with disulfide bonds but also for proteins requiring a reduced state. This versatility provides a more effective strategy for efficient functional protein synthesis with potential applications in biotechnology and therapeutic protein production.

## Materials and Methods

### Materials

KGlu and Mg­(OAc)_2_ were purchased from Sigma-Aldrich and FUJIFILM Wako (Japan), respectively, except where otherwise indicated in figure legends. DTT, TCEP, and EDTA were purchased from Naclalai tesque (Japan). GSH and DTPA were purchased from FUJIFILM Wako. DFP was purchased from Sigma-Aldrich. RNase-free distilled water, DTNB, and PNPP substrate kit were purchased from Thermo Fisher Scientific.

### Preparation of Template DNA for Cell-Free Protein Synthesis

Mature regions of *E. coli* ALP DNA were amplified from *E. coli* genomic DNA by PCR. Human PTP1B (Uniprot no. P18031) DNA was designed using CodHonEditor^22^ based on *E. coli* codon usage and synthesized by Eurofin genomics. The 5′-UTR (5′- GAA­ATT­AAT­ACG­ACT­CAC­TAT­AGG­GAG­ACC­ACA­ACG­GTT­TCC­CTC­TAG­AAA­TAA­TTT­TGT­TTA­ACT­TTA­AGA­AGG­AGA­TAT­ACCA-ORF-3′), containing the T7 promoter and Shine–Dalgarno sequence, and the 3′-UTR (5′-ORF-TAA­TGA­ATA­ACTA­ATCC-3′) were added to all template DNA by PCR. To increase the synthesis efficiency of ALP and PTP1B, Ser-Lys-Tyr was inserted immediately after the first methionine.^9^ The sequences of the template DNA for ALP and PTP1B are shown in Table S1. The amplified DNAs, which were identified by agarose gel electrophoresis, were purified with a PCR purification kit (NucleoSpin Gel and PCR Clean-up (Takara Bio)) and their concentrations were determined by measurement of absorbance at 260 nm.

### Cell-Free Protein Synthesis

The PURE*frex* 2.1 Kit (GeneFrontier, Chiba, Japan) was used as the PURE system reagent for cell-free protein synthesis. For protein synthesis, 5 or 10 μL of the reaction mixture containing 1 ng/μL (PTP1B) or 2 ng/μL (ALP) template DNA and either 2 mM DTT or 4 mM GSH was incubated at 37 °C for 24 h. To assess the effect of chelators, 2 mM DTPA, 2 mM EDTA, or 2 mM DFP was individually added to the reaction mixtures. For solubility analysis, the resulting mixture was centrifuged at 20,000*g* for 30 min at 4 °C, and the supernatants were collected. Synthesized ALP and PTP1B were analyzed by SDS-PAGE under reducing (with β-mercaptoethanol) and nonreducing (without β-mercaptoethanol) conditions as described in the figure legends. Gels were stained with SYPRO Orange (Thermo Fisher Scientific) and visualized using a LuminoGraph imaging system (ATTO, Japan).

### Enzyme Activity Assay

For ALP, the reaction mixture was diluted 20-fold with water. The diluted sample was added to diethanolamine buffer containing PNPP, and the absorbance at 405 nm was measured every 1 min using a Varioskan plate reader (Thermo Fisher Scientific). For PTP1B, the reaction mixture was added to 10 mM HEPES–KOH (pH 7.6) containing PNPP, and the absorbance at 405 nm was measured every 1 min using a Varioskan plate reader. The amount of dephosphorylated PNPP (PNP) calculated from the molar extinction coefficient of PNP (18,000 L·mol^–1^·cm^–1^).

### Quantification of Sulfhydryl Groups Using DTNB

Either 2 mM DTT or 4 mM GSH was incubated at 37 °C for 24 h in the indicated solution as described in the figure legends. Samples (1 μL) containing DTT or GSH were added to 50 μL of 0.2 mM DTNB in 20 mM HEPES–KOH (pH 7.6) and 1 mM EDTA. After incubation at room temperature for 15 min, the absorbance at 412 nm was measured using a Varioskan plate reader. The sulfhydryl content of the prereaction sample dissolved in water was defined as 100%, and subsequent measurements were expressed as relative values based on this reference.

### Reaction in a Glovebox

Reactions containing either 2 mM DTT or 4 mM GSH were prepared in the presence or absence of HKM buffer (100 mM KGlu, 10 mM Mg­(OAc)_2_, and 10 mM HEPES–KOH, pH 7.6) with or without 2 mM DTPA and either inside or outside a glovebox (vinyl anaerobic chamber, Type A, Coy Laboratory Products, Inc.). Stock solutions of 8 mM DTT, 16 mM GSH, 8 mM DTPA, and HKM buffer were prepared outside the glovebox and then transferred into the chamber. All sample solutions were exposed to the glovebox atmosphere for 2 min to equilibrate the headspace gas. Reactions (20 μL each) were assembled on ice in PCR tubes in quadruplicate. The glovebox atmosphere consisted of 98–99% N_2_, 1–2% H_2_, and <30 ppm O_2_. For conditions designated as “outside the glove box”, the assembled samples were taken from the chamber after setup and exposed to the air for 1 min with the lid open to equilibrate the headspace gas. All reactions were incubated at 37 °C for 24 h.

## Supplementary Material



## Notes

Measured absorbance values of PNP in Figure 1D (Figure S1); comparison of potassium and magnesium salts from different suppliers on DTT oxidation (Figure S2); effect of chelators on GSH (Figure S3); effect of DTPA on the synthesis of DHFR (Figure S4); measured absorbance values of PNP in Figure 4B,D (Figure S5); solubility of synthesized ALP and PTP1B (Figure S6); protein synthesis in the presence of TCEP (Figure S7); nucleotide sequences used in this study (Table S1) (PDF)

## Abbreviations

DTT: dithiothreitol
GSH: reduced glutathione
CFPS: cell-free protein synthesis
ALP: alkaline phosphatase
DTNB: 5,5′-dithiobis­(2-nitrobenzoic acid)
KGlu: potassium glutamate
Mg­(OAc)_2_: magnesium acetate
EDTA: ethylenediaminetetraacetic acid
DTPA: diethylenetriaminepentaacetic acid
DFP: deferiprone
PNPP: *p*-nitrophenyl phosphate
