# Supplementary material for: Impact of Reducing Agents on Protein Synthesis in a Reconstituted Cell-Free Protein Synthesis System
Source: ACS Synth Biol. 2026 Feb 17;15(3):1001–7. doi: 10.1021/acssynbio.6c00011 (PMC13010799; doi:10.1021/acssynbio.6c00011)
Supplement: Supplementary file 1 [file sb6c00011_si_001.pdf]

Supporting Information

Impact of reducing agents on protein synthesis in a reconstituted cell-free protein synthesis system

Tomoe Fuse-Murakami, Shohei Terazawa, Riddhi Gondhalekar, Shohei Ito, Seiichi Miyawaki, Yusuke Mizukami, Willan P Salgado, Zening Yang, Kosuke Fujishima, and Takashi Kanamori

List of Contents

Figure S1
Measured absorbance values of PNP in Figure 1D
Figure S2
Comparison of potassium and magnesium salts from different suppliers on DTT oxidation
Figure S3
Effect of chelators on GSH
Figure S4
Effect of DTPA on the synthesis of DHFR
Figure S5
Measured absorbance values of PNP in Figure 4B and 4D
Figure S6
Solubility of synthesized ALP and PTP1B
Figure S7
Protein synthesis in th presence of TCEP
Table S1
Nucleotide sequence used in this study

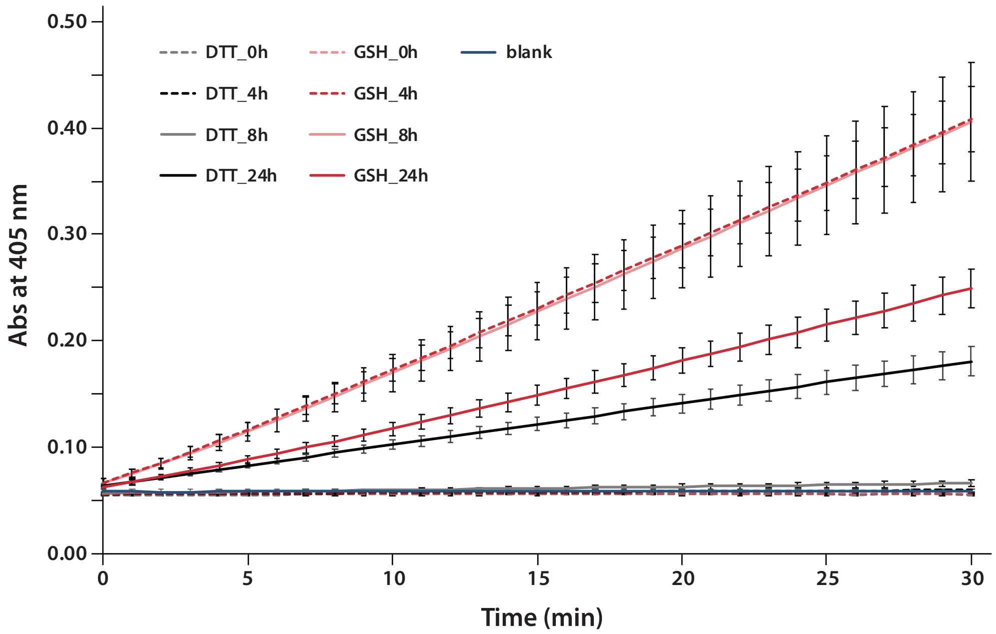

Supplementary Fig. 1.

Measured absorbance values of p-nitrophenol (PNP) in Figure 1D.

Absorbance of PNP was measured every 1 minute at 405 nm using a Varioskan plate reader (Thermo Fisher Scientific). Data are presented as mean ± standard deviation from four independent experiments.

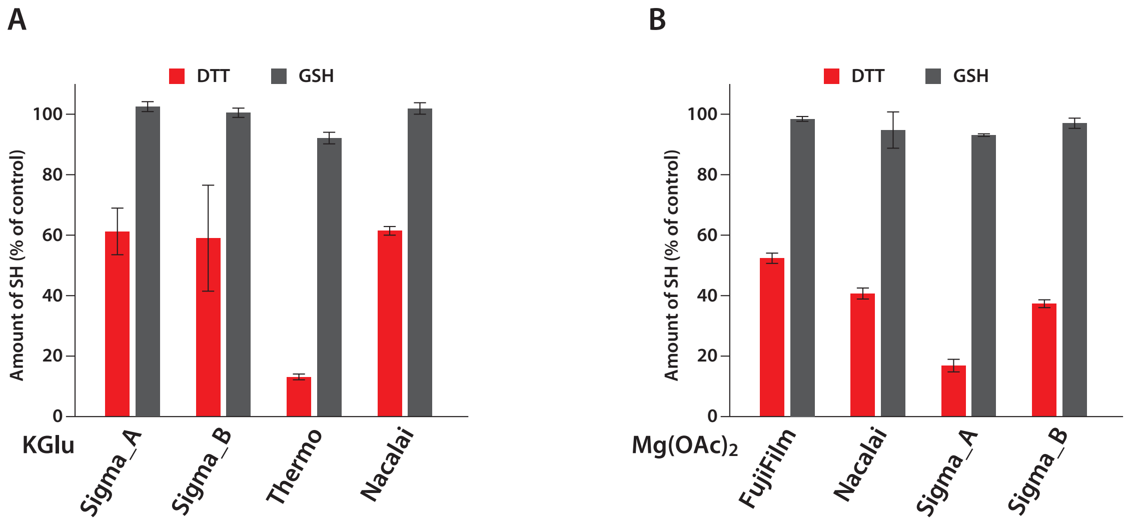

Supplementary Fig. 2.

Comparison of potassium and magnesium salts from different suppliers on DTT oxidation.

DTT or GSH was incubated at 37°C for 24 hours in 100 mM potassium glutamate (KGlu) (A) or 10 mM magnesium acetate ($Mg(OAc)_2$) (B) obtained from the indicated suppliers. After incubation, remaining sulfhydryl groups were measured using DTNB, as described in Materials and Methods. Values are expressed as ratios relative to the initial amount of DTT or GSH in water. Data represent the mean ± standard deviation from four independent experiments. For KGlu, Sigma_A, Sigma_B, Thermo, and Nacalai correspond to Sigma (G1501), Sigma (49601), Thermo Fisher Scientific (A17232-20), and Nacalai tesque (16941-74), respectively. For $Mg(OAc)_2$, FujiFilm, Nacalai, Sigma_A, and Sigma_B correspond to FujiFilm Wako (139-15335), Nacalai tesque (20849-32), Sigma (M5661), and Sigma (M2545), respectively.

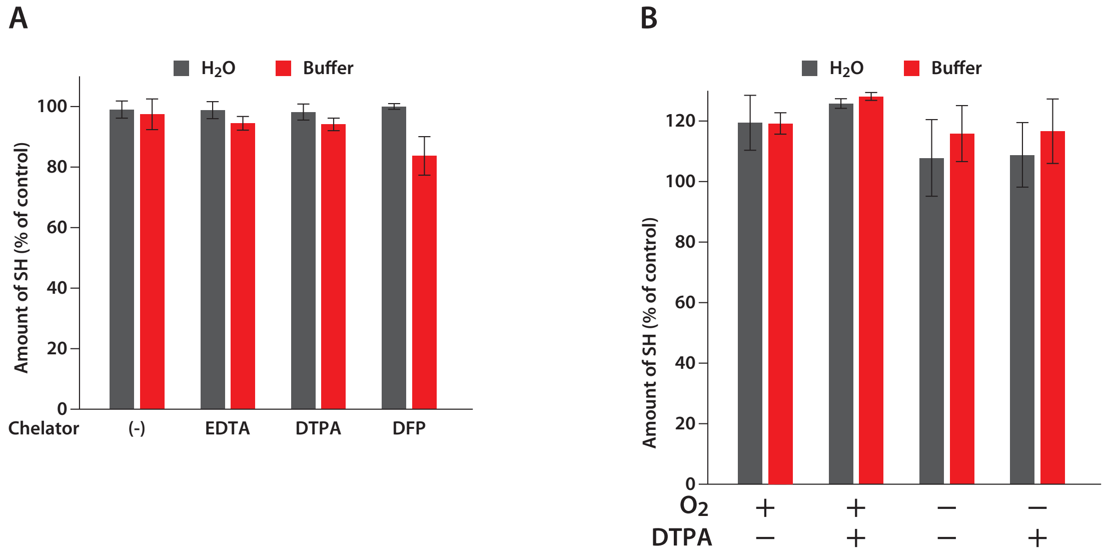

Figure S3

Effect of chelators on GSH. (A) GSH was incubated at 37°C for 24 hours in either water (gray bars) or HKM buffer (red bars), in the presence of the indicated chelators. (B) GSH was incubated at 37°C for 24 hours either inside the glove box (O_2 -) or outside the glove box (O_2 +), with or without DTPA. After incubation, the remaining sulfhydryl groups were measured using DTNB, as described in Materials and Methods. The results are expressed as ratios relative to the initial amount of GSH in water before incubation. Data represent the mean ± standard deviation from three independent experiments.

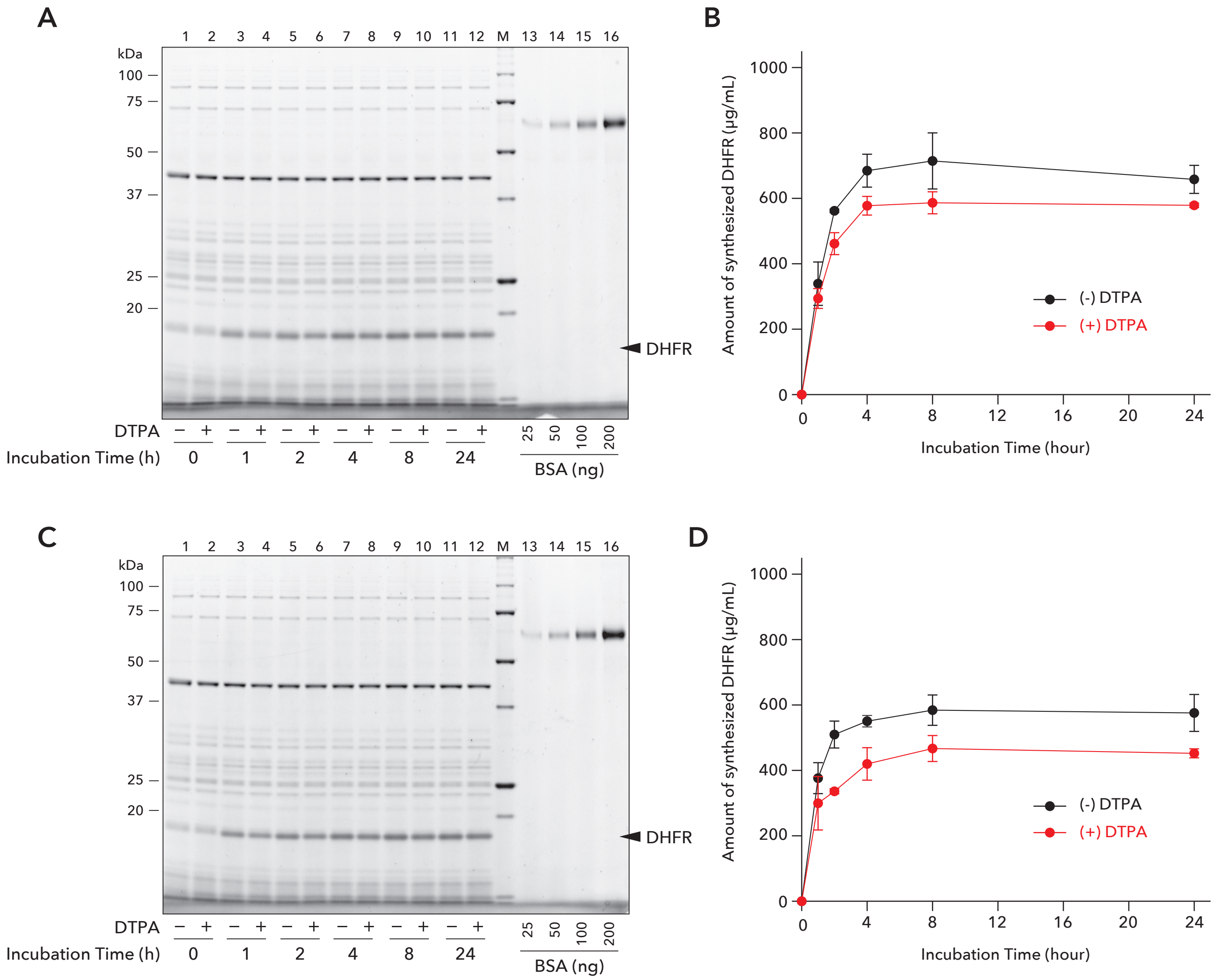

Figure S4

Effect of DTPA on the synthesis of DHFR. *E. coli* DHFR was synthesized using the PURE system in the presence of either DTT (A and B) or GSH (C and D), supplemented with 2 mM DTPA, at 37°C for 24 hours . At the indicated time points, reaction mixtures were collected, and 0.2 µL of each sample was analyzed by SDS-PAGE under reducing conditions. Bovine serum albumin (BSA) was electrophoresed on the same gel for quantificaion. Gels were stained with SYPRO Orange and visualized using LuminoGraph (ATTO) (A and C). The bands of synthesized DHFR were quantified and the amounts were calculated on the BSA standard (B and D). Data are presented as mean ± standard deviation from three independent experiments.

A

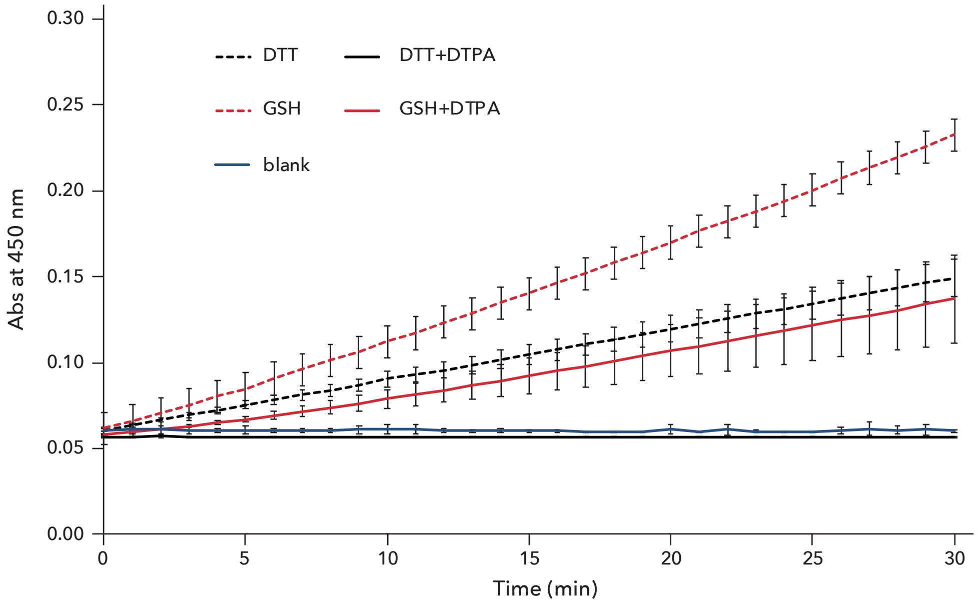

B

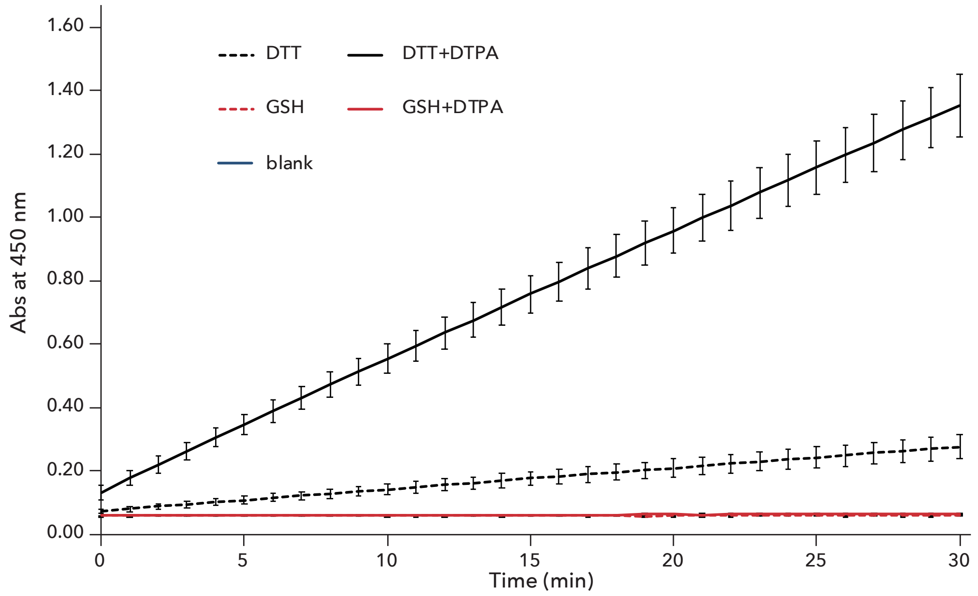

Figure S5

Measured absorbance values of PNP in Figure 4B (A) and 5B (B). Absorbance of PNP was measured every 1 minute at 405 nm using a Varioskan plate reader (Thermo Scientific). Data are presented as mean ± standard deviation from three independent experiments.

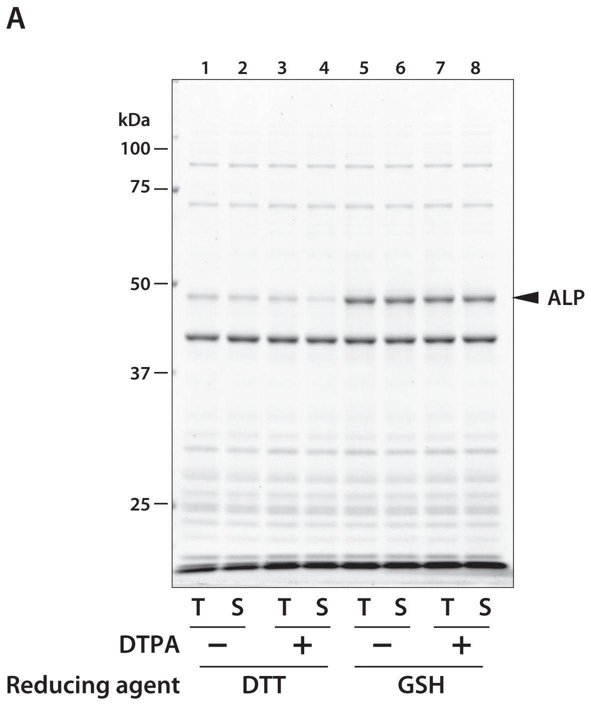

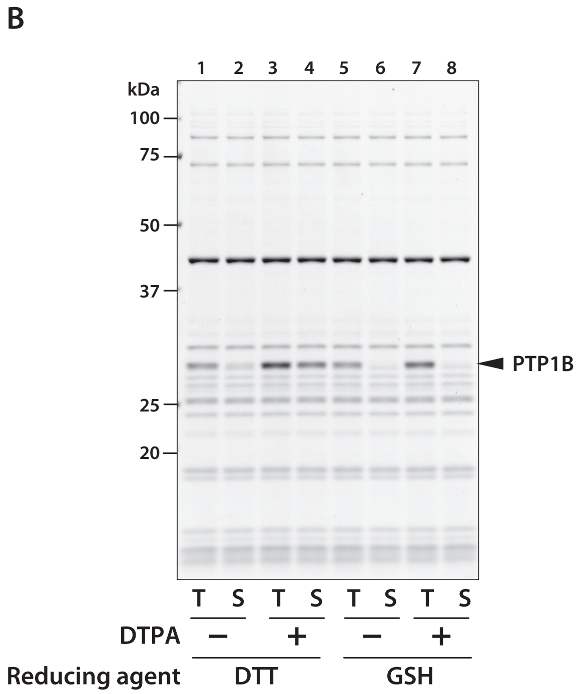

Figure S6

Solubility of synthesized ALP and PTP1B. ALP (A) and PTP1B (B) were synthesized using the PURE system at 37°C for 24 hours in the presence of either DTT or GSH, with or without DTPA. After synthesis, the reaction mixtures were centrifuged at 20,000 × g for 30 minutes, and the supernatants were collected. Both the total reaction mixtures and the supernatants were analyzed by SDS-PAGE under reducing conditions. Gels were stained with SYPRO Orange and visualized using LuminoGraph (ATTO). T and S indicate the total reaction mixture and the supernatant, respectively.

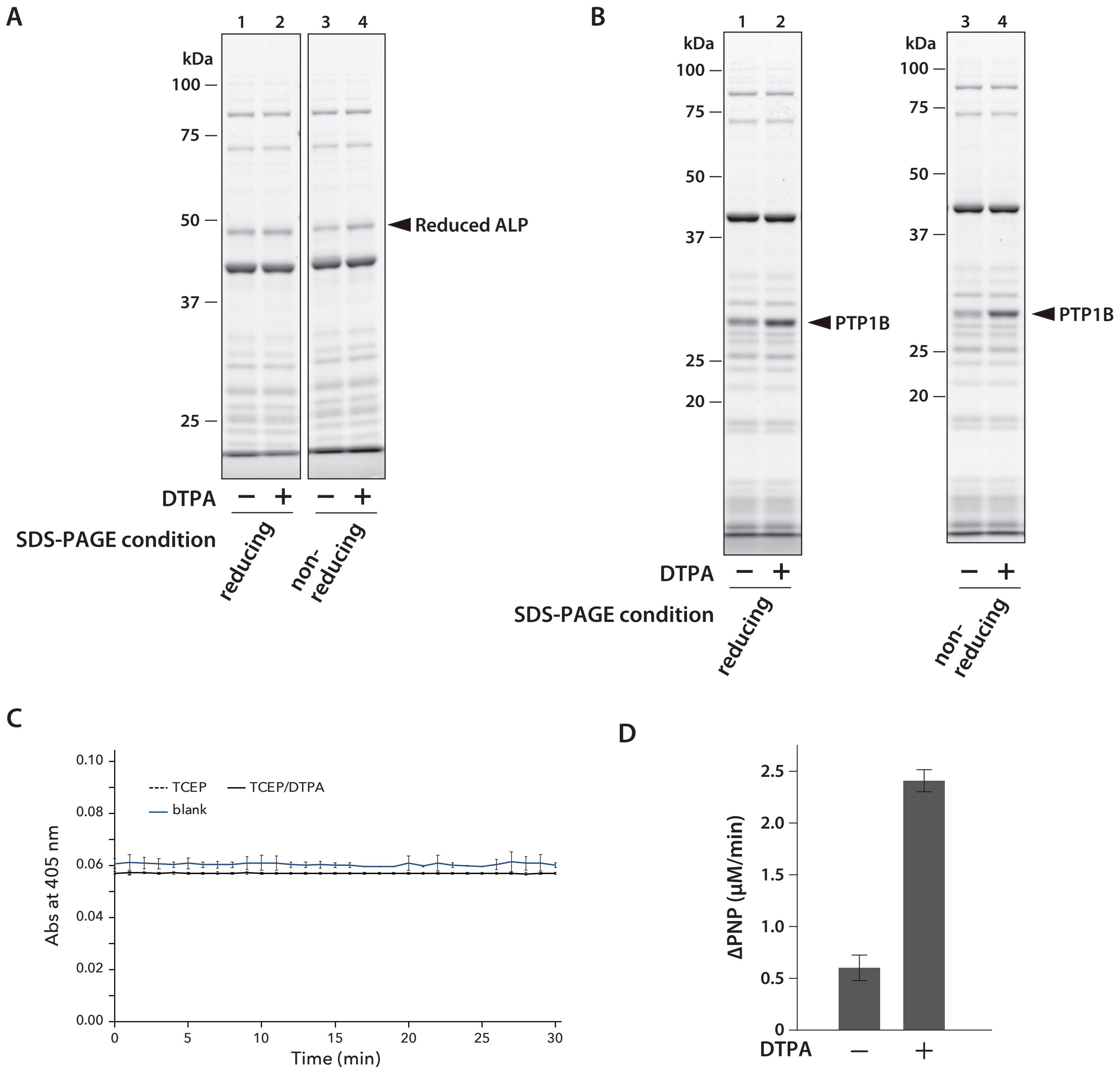

Figure S7

Protein synthesis in th presence of TCEP. ALP (A) and PTP1B (B) was synthesized using the PURE system at 37°C for 24 hours in the presence of TCEP with or without DTPA. Synthesized products were analyzed by SDS-PAGE under reducing and non-reducing conditions. (C) Enzyme activity of the synthesized ALP measured by PNPP phosphatase assay. Absorbance values of PNP was measured every 1 minute at 405 nm using a Varioskan plate reader. Data are presented as mean ± standard deviation from three independent experiments. (D) Enzymatic activity of the synthesized PTP1B was measured by PNPP phosphatase assay. The amount of PNPP hydrolyzed per reaction mixture is shown. Data represent the mean ± standard deviation from three independent experiments.

Table S1. Nucleotide sequence of ALP and PTP1B

Name	Nucleotide sequence
ALP [*1, 2, 3]	gaaattaatacgactcactatagggagaccacaacggtttccctctagaaataattttgtttaactttaagaaggagatataccaATGTCTAAA<br>TATCGGACACCAGAAATGCCTGTTCTGGAAAACCGGGCTGCTCAGGGCGATATTACTGCACCCGGCGGTGCTCGCCGTTTAACGGGTGATCAGA<br>CTGCCGCTCTGCGTGATTCTCTTAGCGATAAACCTGCAAAAAATATTATTTTGCTGATTGGCGATGGGATGGGGGACTCGGAAATTACTGCCGC<br>ACGTAATTATGCCGAAGGTGCGGGCGGCTTTTTTAAAGGTATAGATGCCTTACCGCTTACCGGGCAATACACTCACTATGCGCTGAATAAAAAA<br>ACCGGCAAACCGGACTACGTCACCGACTCGGCTGCATCAGCAACCGCCTGGTCAACCGGTGTCAAAACCTATAACGGCGCGCTGGGCGTCGATA<br>TTCACGAAAAAGATCACCCAACGATTCTGGAAATGGCAAAAGCCGCAGGTCTGGCGACCGGTAACGTTTCTACCGCAGAGTTGCAGGATGCCAC<br>GCCCGCTGCGCTGGTGGCACATGTGACCTCGCGCAAATGCTACGGTCCGAGCGCGACCAGTGAAAAATGTCCGGGTAACGCTCTGGAAAAAGGC<br>GGAAAAGGATCGATTACCGAACAGCTGCTTAACGCTCGTGCCGACGTTACGCTTGGCGGCGGCGCAAAAACCTTTGCTGAAACGGCAACCGCTG<br>GTGAATGGCAGGGAAAAACGCTGCGTGAACAGGCACAGGCGCGTGGTTATCAGTTGGTGAGCGATGCTGCCTCACTGAATTCGGTGACGGAAGC<br>GAATCAGCAAAAACCCCTGCTTGGCCTGTTTGCTGACGGCAATATGCCAGTGCGCTGGCTAGGACCGAAAGCAACGTACCATGGCAATATCGAT<br>AAGCCCGCAGTCACCTGTACGCCAAATCCGCAACGTAATGACAGTGTACCAACCCTGGCGCAGATGACCGACAAAGCCATTGAATTGTTGAGTA<br>AAAATGAGAAAGGCTTTTTCCTGCAAGTTGAAGGTGCGTCAATCGATAAACAGGATCATGCTGCGAATCCTTGTGGGCAAATTGGCGAGACGGT<br>CGATCTCGATGAAGCCGTACAACGGGCGCTGGAATTCGCTAAAAAGGAGGGTAACACGCTGGTCATAGTCACCGCTGATCACGCCCACGCCAGC<br>CAGATTGTTGCGCCGGATACCAAAGCTCCGGGCCTCACCCAGGCGCTAAATACCAAAGATGGCGCAGTGATGGTGATGAGTTACGGGAACTCCG<br>AAGAGGATTCACAAGAACATACCGGCAGTCAGTTGCGTATTGCGGCGTATGGCCCGCATGCCGCCAATGTTGTTGGACTGACCGACCAGACCGA<br>TCTCTTCTACACCATGAAAGCCGCTCTGGGGCTGAAAtaatgaataactaatcc
PTP1B [*1, 3]	gaaattaatacgactcactatagggagaccacaacggtttccctctagaaataattttgtttaactttaagaaggagatataccaATGTCTAAA<br>TATGAAAAAGAATTTGAACAAATTGATAAATCGGGCTCTTGGGCTGCCATCTACCAGGATATTCGACACGAGGCGAGCGACTTCCCGTGCCGTG<br>TAGCAAAGTTGCCAAAGAACAAAAACCGTAACCGCTATCGTGATGTTTCTCCTTTTGACCATTCCCGCATCAAACTGCACCAAGAAGATAATGA<br>CTACATTAACGCTAGTCTTATCAAGATGGAAGAAGCGCAGCGTAGCTACATCCTGACTCAGGGTCCGCTGCCCAACACCTGCGGACATTTCTGG<br>GAGATGGTCTGGGAACAGAAATCTCGGGGTGTGGTTATGTTAAACAGAGTAATGGAAAAAGGCTCCCTGAAATGTGCTCAATATTGGCCGCAGA<br>AGGAGGAAAAAGAAATGATTTTCGAAGATACAAATCTGAAACTCACTCTGATCTCAGAGGACATCAAATCTTACTACACGGTTCGTCAGCTTGA<br>ACTGGAAAACCTGACCACTCAAGAGACCCGCGAAATTCTGCACTTTCACTATACTACGTGGCCGGATTTCGGTGTGCCAGAAAGCCCTGCATCG<br>TTCTTGAACTTTCTGTTCAAGGTACGTGAATCTGGGTCCTTAAGTCCGGAGCATGGCCCGGTTGTCGTTCACTGCAGCGCCGGTATCGGCCGCT<br>CTGGTACCTTCTGTCTGGCTGACACTTGCCTCCTGCTTATGGATAAACGTAAAGACCCGTCCAGCGTGGATATCAAAAAGGTACTGCTGGAAAT<br>GCGTAAATTTCGCATGGGCCTGATTCAGACCGCGGACCAGCTGCGTTTCTCTTACCTGGCAGTTATCGAAGGTGCTAAAtaatgaataactaat<br>cc

1. ORFs are shown by uppercase. T7 promoter and Shine-Dalgarno sequence are underlined and double-lined, respectively.
2. For ALP, only mature region was used.
3. For ALP and PTP1B, the sequence encoding Ser-Lys-Tyr, which was inserted immediately after the first methionine, was shown in red.
